# Intracellular Na^+^ Modulates Pacemaking Activity in Murine Sinoatrial Node Myocytes: An *In Silico* Analysis

**DOI:** 10.3390/ijms22115645

**Published:** 2021-05-26

**Authors:** Stefano Morotti, Haibo Ni, Colin H. Peters, Christian Rickert, Ameneh Asgari-Targhi, Daisuke Sato, Alexey V. Glukhov, Catherine Proenza, Eleonora Grandi

**Affiliations:** 1 Department of Pharmacology, University of California Davis, Davis, CA 95616, USA; hbni@ucdavis.edu (H.N.); aasgari@fas.harvard.edu (A.A.-T.); dsato@ucdavis.edu (D.S.); 2 Department of Physiology and Biophysics, University of Colorado Anschutz Medical Campus, Aurora, CO 80045, USA; colin.peters@cuanschutz.edu (C.H.P.); christian.rickert@cuanschutz.edu (C.R.); catherine.proenza@cuanschutz.edu (C.P.); 3 Department of Medicine, Cardiovascular Medicine, University of Wisconsin Madison School of Medicine and Public Health, Madison, WI 53705, USA; aglukhov@medicine.wisc.edu; 4 Department of Medicine, Division of Cardiology, University of Colorado Anschutz Medical Campus, Aurora, CO 80045, USA

**Keywords:** sodium homeostasis, sodium/potassium pump, sodium/calcium exchanger, sinoatrial node, coupled-clock system, cardiomyocyte, cardiac pacemaking, cardiac arrhythmia, sick sinus syndrome, bistability

## Abstract

*Background*: The mechanisms underlying dysfunction in the sinoatrial node (SAN), the heart’s primary pacemaker, are incompletely understood. Electrical and Ca^2+^-handling remodeling have been implicated in SAN dysfunction associated with heart failure, aging, and diabetes. Cardiomyocyte [Na^+^]_i_ is also elevated in these diseases, where it contributes to arrhythmogenesis. Here, we sought to investigate the largely unexplored role of Na^+^ homeostasis in SAN pacemaking and test whether [Na^+^]_i_ dysregulation may contribute to SAN dysfunction. *Methods*: We developed a dataset-specific computational model of the murine SAN myocyte and simulated alterations in the major processes of Na^+^ entry (Na^+^/Ca^2+^ exchanger, NCX) and removal (Na^+^/K^+^ ATPase, NKA). *Results*: We found that changes in intracellular Na^+^ homeostatic processes dynamically regulate SAN electrophysiology. Mild reductions in NKA and NCX function increase myocyte firing rate, whereas a stronger reduction causes bursting activity and loss of automaticity. These pathologic phenotypes mimic those observed experimentally in NCX- and ankyrin-B-deficient mice due to altered feedback between the Ca^2+^ and membrane potential clocks underlying SAN firing. *Conclusions*: Our study generates new testable predictions and insight linking Na^+^ homeostasis to Ca^2+^ handling and membrane potential dynamics in SAN myocytes that may advance our understanding of SAN (dys)function.

## 1. Introduction

In a healthy individual, each cardiac beat is initiated by the periodic activation of the sinoatrial node (SAN), the primary pacemaker of the heart [1]. The SAN is a complex and heterogeneous structure [2] that consists of a mix of fibroblasts, atrial myocytes, and a subpopulation of specialized myocytes characterized by the peculiar capability of spontaneously firing an action potential (AP), which is the main determinant of SAN pacemaking activity. It is well known that multiple mechanisms forming a “coupled-clock system” are involved in sustaining the automaticity of the spontaneously beating SAN myocytes (SAMs) [3,4]. The first subsystem, called “membrane clock”, encompasses sarcolemmal ion channels and transporters that exhibit voltage- and time-dependent properties and interact nonlinearly to shape AP characteristics. Those include the hyperpolarization-activated cyclic nucleotide-gated channels (carrying the “funny” current I_f_, the dominant driver of early depolarization) [5], the voltage-gated L-type and T-type Ca^2+^ channels (carrying I_CaL_ and I_CaT_, respectively) [6], various voltage-gated Na^+^ and K^+^ channels, and the electrogenic Na^+^/K^+^ ATPase (NKA) and Na^+^/Ca^2+^ exchanger (NCX) [3,4]. NKA consumes ATP to steadily extrude 3 Na^+^ ions and import 2 K^+^ ions (thereby contributing an outward current). In contrast, NCX exchanges one Ca^2+^ ion for 3 Na^+^ ions and, depending on the electrochemical gradients of Na^+^ and Ca^2+^, results in either inward or outward current (with the former mode being predominant during the AP). The second subsystem, called “Ca^2+^ clock”, refers to the mechanisms responsible for regulating intracellular Ca^2+^ concentration ([Ca^2+^]_i_), including the release of Ca^2+^ from and reuptake into the sarcoplasmic reticulum (SR). In particular, local spontaneous Ca^2+^ releases occurring periodically during diastole activate inward NCX current (in Ca^2+^-extrusion mode), contributing to diastolic depolarization by directly coupling Ca^2+^ handling with AP dynamics [3,4]. Coupling between the membrane and Ca^2+^ clocks is also modulated by other [Ca^2+^]_i_-dependent processes, such as Ca^2+^-dependent inactivation (CDI) of I_CaL_ [7], and is bidirectional, in that AP morphology and duration also affect cytosolic and SR Ca^2+^ levels by shaping Ca^2+^ influx and efflux. Disruption of the mechanisms influencing the coupled-clock system impairs regulation of SAM automaticity and can compromise SAN function leading to pathologic phenotypes, including bradycardia, tachy-brady arrhythmias, and sinus arrest [8]. While genetic mutations can cause SAN dysfunction [6,9,10], this condition, also called “sick sinus syndrome”, is associated with more common widespread diseases like heart failure (HF) and atrial fibrillation [11,12], or aging [13]. Despite progress made with animal studies in the last decades to unravel the pathophysiological mechanisms underlying SAN dysfunction, our understanding of the disease in humans and treatment options for patients are limited [8], whereby implantation of an electronic pacemaker is still the most common solution [10].

One understudied aspect of the regulation of SAN electrophysiology is the role of intracellular Na^+^ ([Na^+^]_i_) homeostasis. [Na^+^]_i_ has emerged as a key regulator of cardiac excitation-contraction coupling in ventricular and atrial myocytes [14,15,16], in that it controls not only cardiac inotropy by affecting the ability of NCX to extrude Ca^2+^, but also affects cardiac excitability and AP duration by modulating voltage-gated Na^+^ channels [17,18] and electrogenic NKA and NCX currents [19,20,21]. Indeed, in ventricular myocytes from HF rabbits and patients, excessive Na^+^ accumulation limits NCX-mediated Ca^2+^ extrusion, possibly leading to diastolic dysfunction and increased propensity for Ca^2+^-induced triggered arrhythmias [20,21,22,23,24,25]. Unlike [Ca^2+^]_i_, which shows large changes from ~100 nM to ~1 µM within each cardiac cycle (i.e., a few hundreds of milliseconds) [26], the balance between Na^+^ influx and efflux mechanisms results in a relatively stable [Na^+^]_i_ from beat to beat, with changes requiring many seconds to minutes. Indeed, slow changes in [Na^+^]_i_ have been shown to underlie intermittent arrhythmias in both atrial and ventricular myocytes [27,28]. While these Na^+^ channels and transport mechanisms are mostly maintained in SAMs, distinct SAN AP characteristics and transmembrane ion channel repertoire may uniquely shape Na^+^ in these cells. Furthermore, the effect of alterations in Na^+^ homeostasis on SAM automaticity is largely unexplored, nor is it known whether changes in [Na^+^]_i_ are involved in SAN remodeling and dysregulation observed, e.g., in HF patients [11]. Interestingly, abnormalities in Na^+^ influx and extrusion pathways, via either NCX knockout or ankyrin-B loss of function (which disrupts localization and expression of NCX and NKA) lead to irregular SAN activity, including tachy-brady arrhythmias and increased heart rate variability [29,30,31,32,33].

Here, we aim to quantitatively assess the impact of [Na^+^]_i_ changes on SAM automaticity and to test whether Na^+^ dysregulation may contribute to SAN dysfunction. To pursue this goal, we developed a dataset-specific computational model of the murine SAM and simulated the effects of blocking NKA and NCX (i.e., the major mechanisms of Na^+^ removal and influx, respectively). We demonstrate that Na^+^ homeostasis dynamically regulates SAM electrophysiology, whereby Na^+^ changes can lead to an array of arrhythmogenic phenotypes, including tachycardia, bursting behavior, and complete loss of automaticity. Our study provides new insight into the link between [Na^+^]_i_, [Ca^2+^]_i_, and AP dynamics in SAMs and generates new testable predictions that may advance our understanding of cardiac pacemaking function and dysfunction.

## 2. Results

### 2.1. A Computational Model of Murine SAMs Well Recapitulates a Broad Experimental Dataset

**Figure 1:**
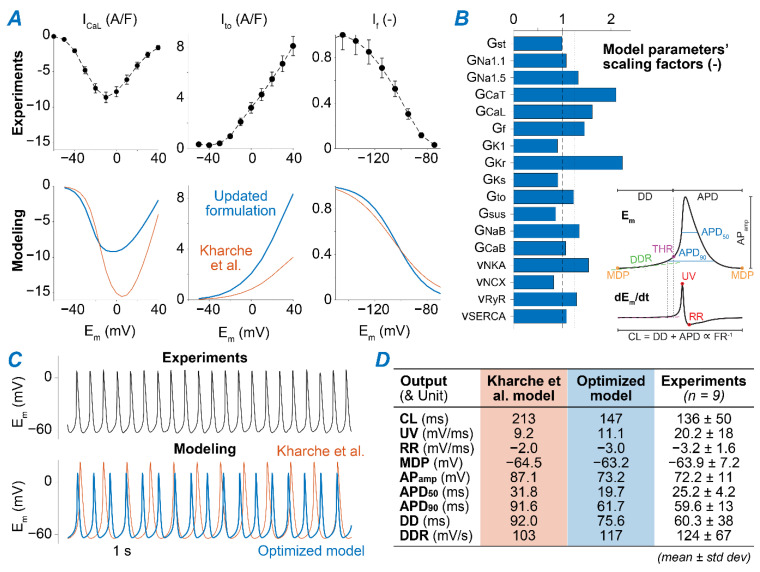
Reparameterization of the Kharche et al. model of the murine SAM. (**A**) Experimental (top panels) and simulated (bottom panels) voltage-dependence of peak I_CaL_ [24], peak I_to_ [25], and I_f_ availability [26]. (**B**) Parameter scaling factors yielded by the global optimization process aimed at minimizing the differences between average measured and simulated AP characteristics (shown in the schematic in the inset). Definition of both model parameters and AP characteristics is reported in Appendix A. (**C**) Time course of membrane voltage in a representative experimental trace and in simulations performed with the original Kharche et al. model (orange) and our optimized dataset-specific reparameterization (blue). (**D**) Comparison between experimentally measured AP characteristics [23] and the outputs predicted with original and optimized models.

**Figure 2:**
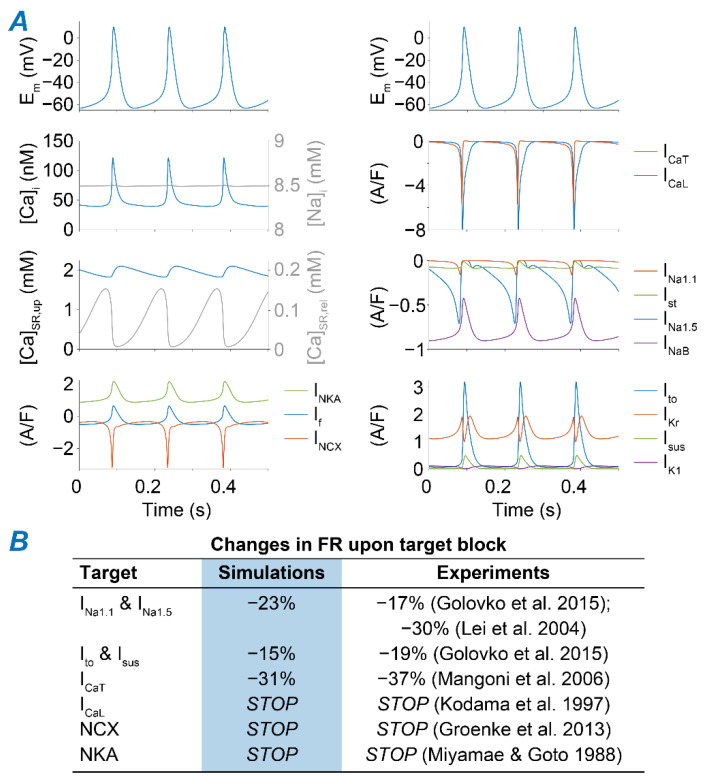
Properties of the newly developed dataset-specific model of the murine SAM. (**A**) Time course of AP, Ca^2+^ and Na^+^ concentrations, and main ion currents in the optimized model. (**B**) Simulated and experimentally observed effects on FR induced by complete block of I_Na1.1_ and I_Na1.5_ [28,29], I_to_ and I_sus_ [29], I_CaT_ [30], I_CaL_ [31], NCX [32], and NKA [33]. “Stop” indicates interruption of spontaneous firing activity.

We simulated the murine SAM AP using the published model developed by Kharche et al. [34], updated to more closely recapitulate our experimental datasets. First, we adjusted the formulation of several ionic currents based on our measurements of I_CaL_ [35], transient and steady-state outward K^+^ currents (I_to_ and I_sus_) [36], and I_f_ [37] (Figure 1A). Then, we applied a global optimization method [38] to scale selected model parameters (Table A1) to match measured AP characteristics (Figure 1B and Table A2) [39,40]. The resulting optimized model reproduces AP waveform properties that closely resemble our experimental results (Figure 1C,D and Figure 2A). As an additional validation step, we confirmed that the model reproduces the reported effects of selectively blocking individual ion channels and transporters on SAM firing rate (FR, Figure 2B) [41,42,43,44,45,46].

### 2.2. NKA and NCX Inhibition Can Both Boost and Disrupt SAM Automaticity

**Figure 3:**
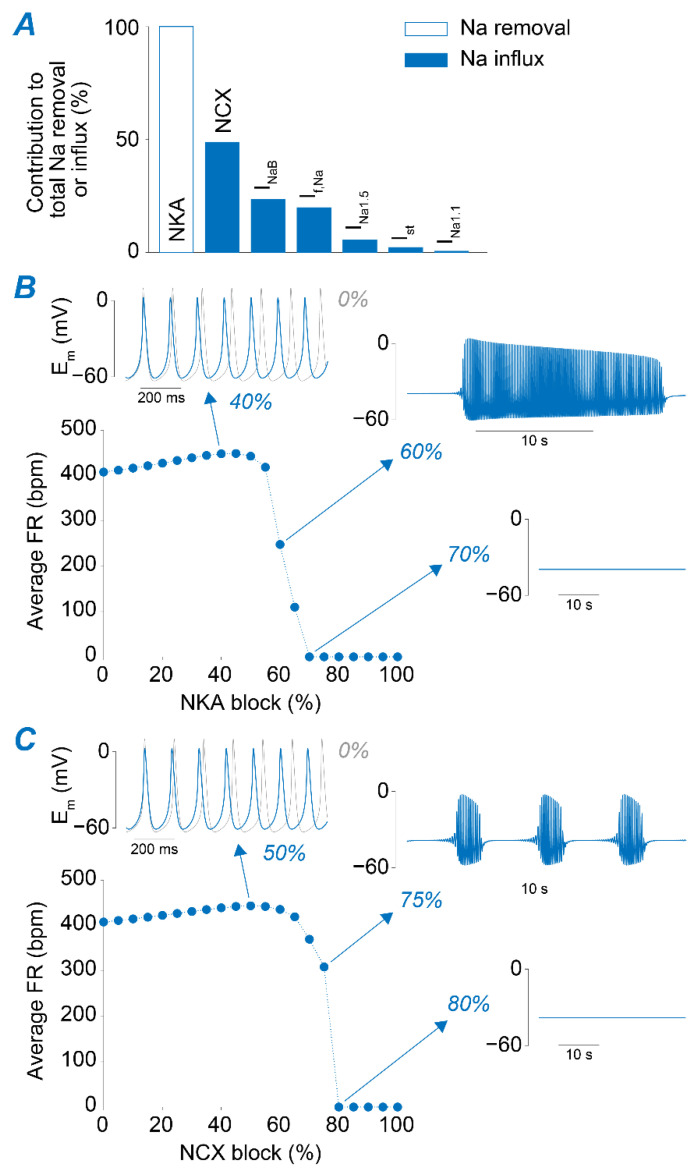
NKA and NCX reductions can both enhance SAM automaticity and impair pacemaking activity depending on the degree of block. (**A**) Bar graph showing the relative contribution of NKA, NCX, I_Na1.1_, I_Na1.5_, I_st_, I_NaB_, and I_f_ to total Na^+^ influx and removal during a beat cycle in the optimized murine SAM model. (**B**) Average FR upon simulation of various extents of NKA block. Insets show voltage traces obtained simulating 40%, 60%, and 70% block. (**C**) Average FR upon simulation of various extents of NCX block. Insets show voltage traces obtained simulating 50%, 75%, and 80% block. Thin gray traces in insets correspond to the voltage traces simulated in the absence of the block.

Regulation of [Na^+^]_i_ depends on the balance between Na^+^ influx and efflux through the sarcolemmal membrane during the beat cycle. Figure 3A depicts the predicted contribution of various mechanisms for Na^+^ removal (NKA) and Na^+^ influx, mediated by the voltage-gated TTX-sensitive, TTX-insensitive, and sustained Na^+^ currents (I_Na1.1_, I_Na1.5_, and I_st_, respectively), a passive Na^+^ leak (I_NaB_), I_f_ (which carries both Na^+^ and K^+^), and NCX [34]. In the model, NCX constitutes the main Na^+^ entry pathway, accounting for ~50% of the total Na^+^ influx, whereas NKA is the only Na^+^ extrusion pathway. Thus, we manipulated their respective maximal transport rates (v_NKA_ and v_NCX_) to produce substantial changes in Na^+^ homeostasis and investigate the effects induced by Na^+^ accumulation and depletion on SAN automaticity. Interestingly, despite opposite effects on [Na^+^]_i_, we observed similar phenotypes when progressively increasing the degree of the block of each transporter (Figure 3B,C). Reducing NKA or NCX function up to ~60 and 50%, respectively, has a slight positively chronotropic effect. However, higher degrees of block lead to a decrease in FR and irregularities in pacemaking function, in which the SAM capability of firing APs is periodically lost and restored. Further reductions in NKA (≥70%) or NCX (≥80%) eventually lead to permanent loss of SAM automaticity, in agreement with experimental observations [45,46]. We analyzed these results and performed simulations to dissect the mechanisms underlying the observed phenotypes, as detailed in the following sections.

### 2.3. Na^+^ Accumulation Induced by NKA Impairment Reduces SAM Automaticity via Ca^2+^ Overload and Excessive Ca^2+^-Dependent I_CaL_ Inactivation

**Figure 4:**
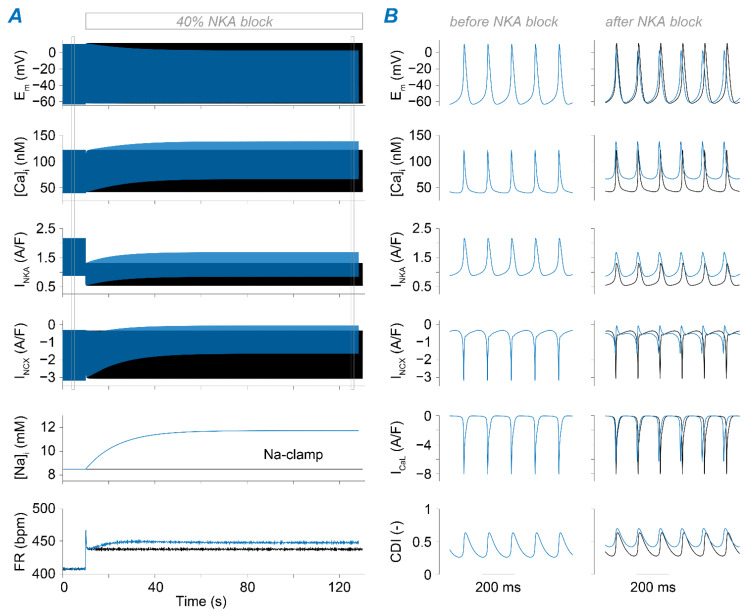
Mild block of NKA has positive chronotropic and isotropic effects. (**A**) Time course of membrane potential, [Ca^2+^]_i_, NKA current, [Na^+^]_i_, and FR predicted upon sudden 40% block of NKA maximal transport rate (at *t* = 10 s). Black traces are obtained clamping [Na^+^]_i_ to the initial value, while blue traces are obtained simulating the normal condition in which [Na^+^]_i_ is free to change. (**B**) Comparison between the time course before applying the block and at the end of the simulation for membrane potential, [Ca^2+^]_i_, NKA current, NCX current, I_CaL_, and its Ca^2+^-dependent inactivation. CDI values were calculated from the state variable *Fca*, representing the gate describing CDI in the Hodgkin-Huxley type I_CaL_ model in the Kharche et al. framework (CDI = 1 − *Fca*, with *Fca* varying from 0 to 1).

**Figure 5:**
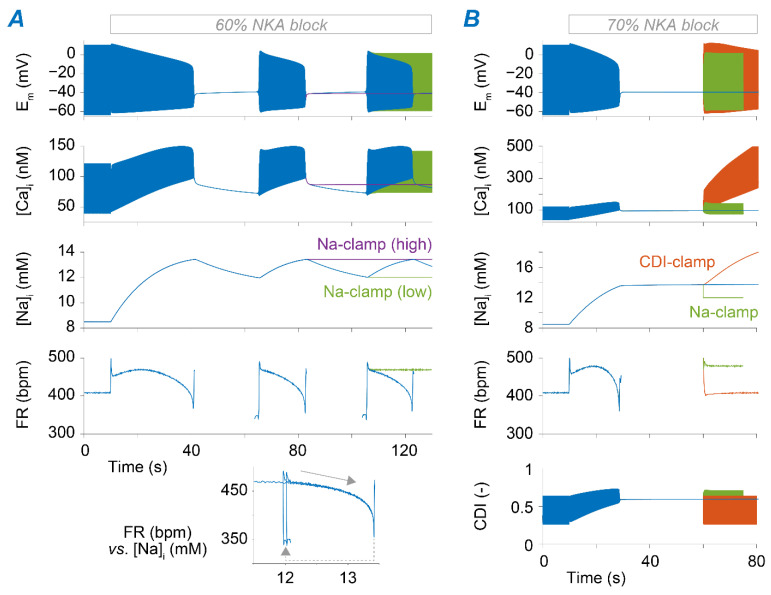
Excessive Na^+^ accumulation and consequent Ca^2+^ overload upon NKA block impair SAM automaticity. (**A**) Time course of membrane potential, [Ca^2+^]_i_, [Na^+^]_i_, and FR simulated upon 60% block of NKA (at *t* = 10 s). Blue traces are obtained simulating the baseline condition in which [Na^+^]_i_ is free to change. Purple and green traces are obtained by clamping [Na^+^]_i_ to either the maximal or the minimum values in the blue trace. The inset shows the FR-[Na^+^]_i_ phase plot obtained during the bistable regime. (**B**) Time course of membrane potential, [Ca^2+^]_i_, [Na^+^]_i_, FR, and CDI of I_CaL_ obtained upon simulation of 70% block of NKA (at *t* = 10 s). Green traces are obtained by clamping [Na^+^]_i_ to 12 mM after *t* = 60 s; orange traces are obtained by imposing (after *t* = 60 s) the values of CDI predicted before NKA block; blue traces are obtained simulating the model without any constrain on [Na^+^]_i_ or CDI.

The time course of SAM response to NKA blockade reveals both instantaneous and gradual changes in SAM electrophysiology (Figure 4 and Figure 5). This is due to the abrupt reduction in the electrogenic NKA current that alters voltage dynamics and thus FR instantaneously, and the slower consequent increase in Na^+^, which modulates Ca^2+^ homeostasis and AP dynamics. When reducing v_NKA_ by 40%, decreased outward current accelerates diastolic depolarization and suddenly enhances SAM automaticity (Figure 4; after a transient spike that fades in a few beats). Then, [Na^+^]_i_ accumulates over time and leads to further enhancement in SAM automaticity. This further FR increase is due to [Na^+^]_i_-mediated increase in [Ca^2+^]_i_ and consequently increased NCX activity during diastole. In fact, when we repeated the simulation with [Na^+^]_i_ clamped at the initial value to prevent its elevation, the slow FR adaptation phase is prevented. Our simulations also predict a reduced steady-state AP amplitude due to increased I_CaL_ CDI limiting the AP upstroke (Figure 4).

As shown in Figure 3B, the steady-state response leads to very different outcomes upon a higher degree of NKA block. When reducing v_NKA_ by 60%, the model enters a bistable regime in which the SAM exhibits intermittent firing activity (Figure 5A). The switch between these two states is determined by slow changes in [Na^+^]_i_ that accumulates during the active phase and diminishes during the pauses. To demonstrate that this arrhythmic phenotype is caused by Na^+^ accumulation and depletion, we ran additional simulations in which [Na^+^]_i_ was clamped to the maximal or minimal Na^+^ levels (~13.5 and ~12 mM, respectively) seen during the oscillations. Indeed, model results predict that the bursting behavior is suppressed if Na^+^ is kept constant, whereby Na^+^ elevation (or depletion) can permanently interrupt (or restore) regular AP firing (Figure 5A). Permanent loss of automaticity is observed when the NKA block is ≥70%. In this case, Na^+^ levels increase due to limited extrusion, and the SAM membrane potential stabilizes at ~−40 mV, similarly to the value reported in rabbit multicellular SAN preparations [46]. In these conditions, regular firing activity could be restored by clamping [Na^+^]_i_ in our model to a lower level (12 vs. ~14 mM, Figure 5B).

To identify the mechanism linking Na^+^ overload to loss of AP firing, we analyzed the changes in currents and transporters modulated by [Na^+^]_i_, either directly or indirectly (e.g., via Ca^2+^ overload). We found that Na^+^-dependent Ca^2+^ accumulation increases CDI of I_CaL_ to the extent that it hampers a current essential for generating the AP [44,47]. Indeed, our simulations reveal that firing activity can be re-initiated (even with high Na^+^ load) by restoring CDI to the levels seen before perturbing NKA function (Figure 5B).

### 2.4. Ca^2+^ Accumulation Induced by NCX Impairment Reduces SAM Automaticity via Excessive Ca^2+^-Dependent I_CaL_ Inactivation

**Figure 6:**
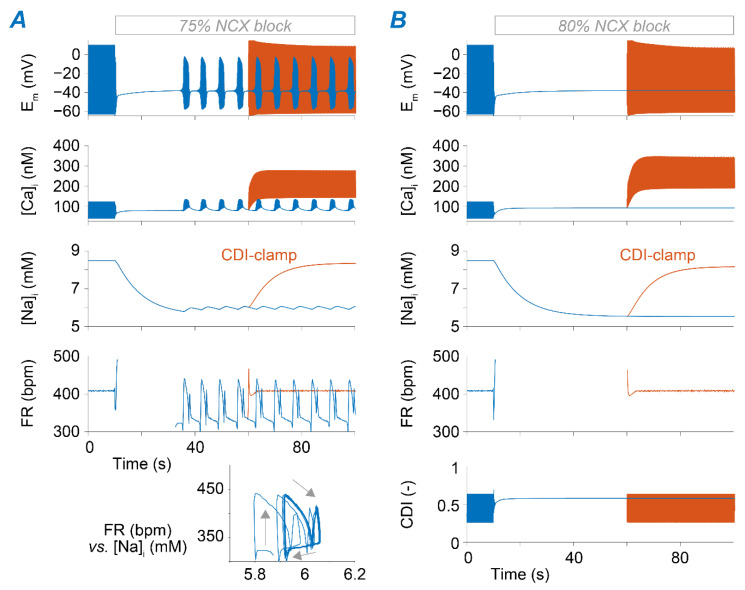
Ca^2+^-dependent I_CaL_ inactivation upon NCX block impairs SAM automaticity. (**A**) Time course of membrane potential, [Ca^2+^]_i_, [Na^+^]_i_, and FR upon simulation of 75% block of NCX (at *t* = 10 s). The inset shows the FR-[Na^+^]_i_ phase plot obtained when the model enters the bursting regime. (**B**) Time course of membrane potential, [Ca^2+^]_i_, [Na^+^]_i_, FR, and CDI of I_CaL_ upon simulation of 80% block of NCX (at *t* = 10 s). In both panels, orange traces are obtained by imposing (after *t* = 60 s) the values of CDI predicted before NCX block; blue traces are obtained simulating the model without any constrain on CDI.

Despite the opposing roles of NKA and NCX in regulating cellular Na^+^ loading, the block of these transporters has similar consequences in terms of changes in FR (Figure 3). The next set of simulations aimed at identifying similarities and differences in the underlying mechanisms. We found that positive chronotropy induced by reducing v_NCX_ up to 60% is primarily mediated by increased [Ca^2+^]_i_ that enhances NCX activity during diastole and limits I_CaL_ (via CDI), leading to a lower AP peak (Figure S1). The moderate [Na^+^]_i_ decrease predicted in this case weakly counteracts Ca^2+^ accumulation and contributes to the chronotropic effect by slightly decreasing outward NKA current and facilitating diastolic depolarization (Figure S1). Upon 75% reduction of v_NCX_, the cell enters a bistable regime characterized by periodic transitions between the active (firing) and inactive states (Figure 6A), accompanied by small oscillations in [Na^+^]_i_. Once again, disruption of regular pacemaking is due to excessive CDI of I_CaL_ induced by intracellular Ca^2+^ accumulating during the active phase. When simulating ≥80% v_NCX_ reduction, SAM automaticity is permanently lost (Figure 6B), in agreement with observations in murine SAMs lacking NCX [45]. As previously shown for NKA block, disruption of SAM automaticity is primarily determined by Ca^2+^ overload, which hampers I_CaL_ via excessive CDI (Figure 6B).

### 2.5. Concomitant NKA, NCX, and I_CaL_ Block Increases the Susceptibility to Pacemaking Dysfunction

**Figure 7:**
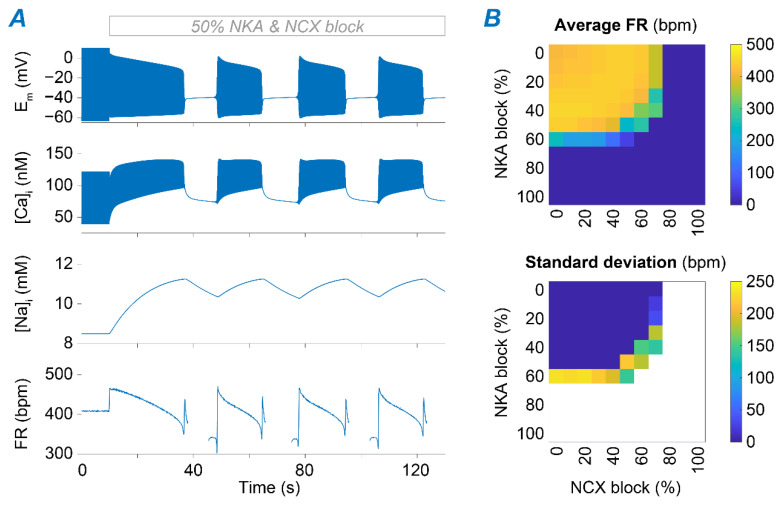
Combining NKA and NKA block facilitates disruption of SAM automaticity. (**A**) Time course of membrane potential, [Ca^2+^]_i_, [Na^+^]_i_, and FR upon simulation of combined 50% block of NKA and NCX. (**B**) Average FR (top panel) and its standard deviation (bottom panel) were assessed upon combinations of concomitant NKA and NCX block.

We demonstrated that individual blocks of NKA and NCX could impair SAM automaticity. Interestingly, however, concomitant downregulation of NKA and NCX has been observed in mice lacking functional ankyrin-B [31,32] and could be a key contributing factor to the emerging irregularities in SAN function. We hypothesized that impairment in both transporters could act synergistically, whereby smaller concomitant reductions in v_NKA_ and v_NCX_ are required to disrupt SAM firing activity compared to those needed when NKA (≥60%) and NCX (≥75%) are altered individually. To test our hypothesis, we simulated various combinations of NKA and NCX block, ranging from normal function to complete block, and assessed the impact on average FR and FR variability (Figure 7). As hypothesized, our simulations predicted developing irregular pacemaking activity, including bursting and permanent loss of automaticity, with a combined ≥50% reduction of v_NKA_ and v_NCX_.

**Figure 8:**
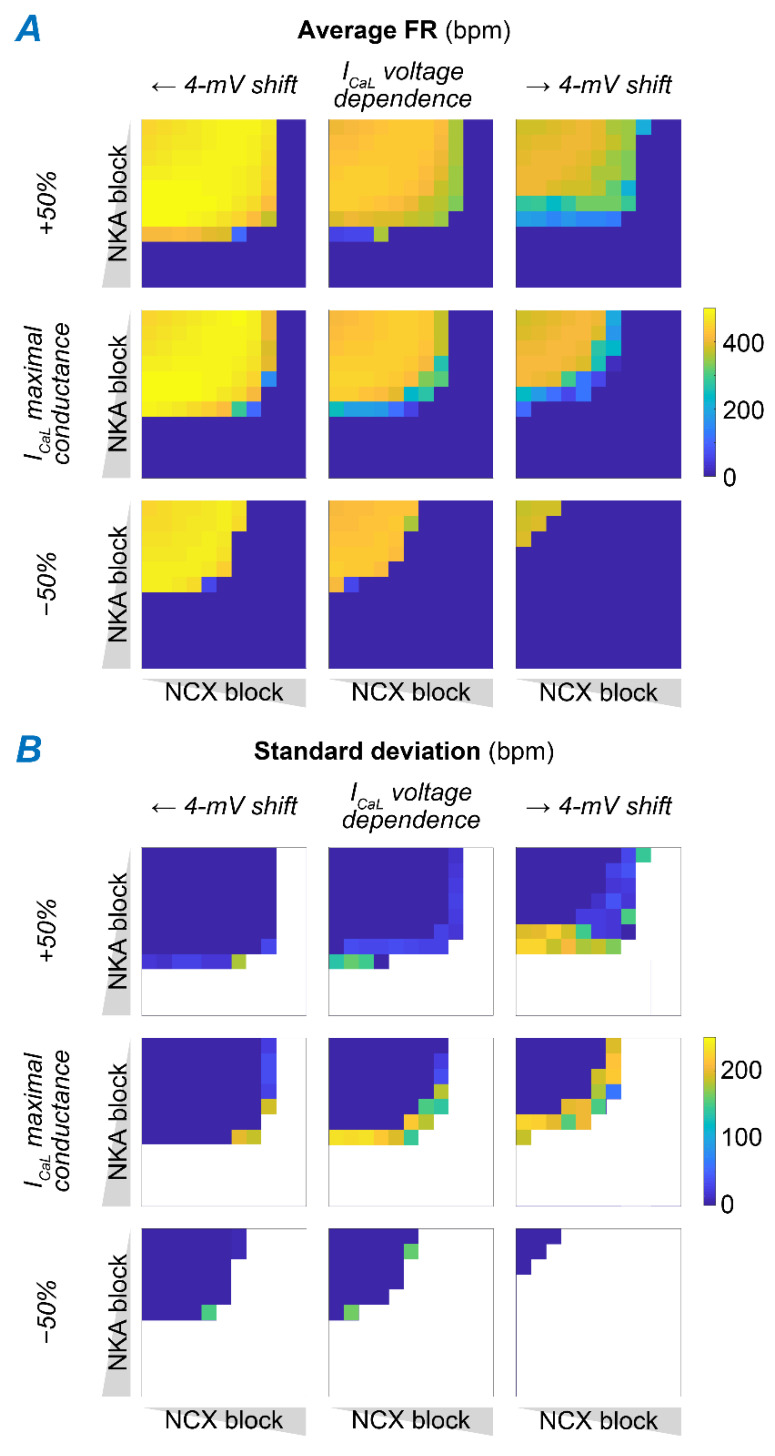
Increased G_CaL_ and leftward shift of I_CaL_ voltage-dependence counteracts disruption of SAM automaticity induced by combined NKA and NCX block. Average FR (**A**) and FR standard deviation (**B**) were assessed upon combinations of concomitant NKA and NCX block with normal or altered I_CaL_ function. Changes in I_CaL_ include modulation of maximal conductance (±50%) and shifts in the voltage-dependence of activation and inactivation (±4 mV).

We previously described the key role of I_CaL_ in the generation of SAM APs, and the deleterious consequences of increasing CDI (Figure 5 and Figure 6). Notably, I_CaL_ was found reduced in dysfunctional SAMs from both NCX knockout and ankyrin-B-deficient mice [29,32], potentially due to a compensatory mechanism to reduce Ca^2+^ load [48]. To assess how changes in I_CaL_ influence SAM response in the face of combined NKA and NCX block, we expanded the parametric analysis in Figure 7 by simulating the effects of variations in I_CaL_ maximal conductance (G_CaL_) and voltage-dependent gating (Figure 8). Our results show that the region in which regular SAM firing activity is preserved shrinks as G_CaL_ is reduced, suggesting that even smaller perturbations of NKA and NCX function could result in SAM dysfunction in the case of I_CaL_ downregulation. Similarly, we observed that the region with irregular firing activity expands as voltage-dependence of I_CaL_ is shifted toward more positive potentials (i.e., to increase its voltage threshold of activation).

## 3. Discussion

### 3.1. Summary of the Results

Intracellular Na^+^ homeostasis is critical in regulating cardiac electrophysiology and contraction in atrial and ventricular myocytes [14,15]. Elevated cardiomyocyte [Na^+^]_i_ has been reported in diseases such as HF and diabetes [16], contributing to arrhythmogenesis and impaired relaxation. SAN dysfunction is often associated with these diseases, but the role of [Na^+^]_i_ in the regulation of cardiac pacemaking is largely unexplored in normal physiology, let alone in disease. Here, we sought to investigate how Na^+^ homeostasis affects SAM automaticity and to test whether [Na^+^]_i_ dysregulation may contribute to SAN dysfunction. We simulated mouse SAM APs using the Kharche et al. model [34], fitted to our experimental dataset recently collected in mice at a physiologic temperature [39] (Figure 1). We showed that disrupting Na^+^ homeostasis by reducing NKA or NCX function leads to an array of phenotypes, including enhanced FR, bursting behavior, and complete loss of automaticity (Figure 3). Our model-based analysis revealed that these behaviors are due to the coupling of Na^+^ homeostasis to Ca^2+^ handling (via NCX) and to membrane potential dynamics (via I_CaL_ CDI) in SAMs (Figure 5 and Figure 6). We also found that NKA and NCX block displays a synergistic pro-arrhythmic effect (Figure 7), and disruption of SAM automaticity is also facilitated by downregulation of I_CaL_ (Figure 8).

### 3.2. Feedback of Na^+^ and Ca^2+^ Signals and Membrane Clock Dynamically Modulates SAM Automaticity

**Figure 9:**
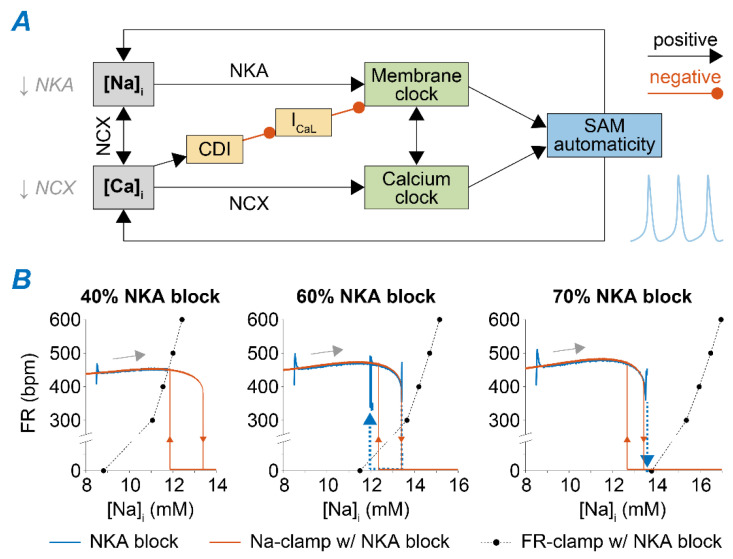
Feedback of Na^+^ and Ca^2+^ signals and membrane clock modulates SAM automaticity. (**A**) Schematic of the relationships linking Na^+^ and Ca^2+^ signaling and SAM automaticity. (**B**) FR-[Na^+^]_i_ phase plots showing FR and [Na^+^]_i_ values predicted over time upon 40%, 60%, and 70% NKA block (blue lines), during quasi-steady-state [Na^+^]_i_-clamp simulations in which [Na^+^]_i_ is slowly (i.e., 0.33 mM/min) increased from 8 to 17 mM and then reduced to 8 mM (orange lines), and at steady-state in voltage-clamp simulations in which the FR is controlled (dotted black lines with dots).

Our results reveal complex interactions of intracellular Na^+^ homeostasis, Ca^2+^ handling, and AP dynamics in SAMs (Figure 9A). At baseline, modest increases in Ca^2+^ and Na^+^ loading enhance FR. Mild inhibition of NKA leads to enhanced automaticity via a direct effect on membrane dynamics (due to a reduced outward current in diastole) and a Na^+^-mediated increase in Ca^2+^ load and consequent NCX increase that accelerates the Ca^2+^ clock. Modest NCX inhibition leads to opposite and smaller changes in [Na^+^]_i_ (Figure S2), but the impaired Ca^2+^ extrusion favors Ca^2+^ accumulation and results in a comparable chronotropic effect. Strong NKA and NCX function reductions lead to excessive Ca^2+^ accumulation, which increases CDI and prevents I_CaL_ and AP firing. For intermediate degrees of NKA and NCX block, SAMs display intermittent firing activity, in which slow changes in [Na^+^]_i_ and diastolic [Ca^2+^]_i_ (which increase during firing and decrease during the pause) drive the switch between predominantly positive or negative coupling between increased Ca^2+^ loading and coupled membrane and Ca^2+^ clock. In the case of the 60% NKA block, the sudden decrease in NKA causes slow Na^+^ accumulation. This increased [Na^+^]_i_ in turn outwardly shifts the NKA current and causes FR to increase but also increases Ca^2+^ loading (due to reduced NCX) and favors CDI. Thus, negative feedback exists between the slow Na^+^ dynamics and the fast [Ca^2+^]_i_-FR subsystem. In phase plots of FR and [Na^+^]_i_, the fast [Ca^2+^]_i_-FR subsystem shows bistable FR (FR nullcline, orange lines in Figure 9B, central panel). The slow [Na^+^]_i_ variable increases monotonically with FR ([Na^+^]_i_ nullcline, black dots). The intersection of these two nullclines, which identifies the system’s fixed point, does not occur in either stable branches of the FR nullcline but crosses the unstable region (Figure 9B, central panel). Thus, the system oscillates, and the FR-Na^+^ phase plot forms a hysteresis loop, corresponding to the quasi-periodic FR fluctuations (i.e., bursting) observed at the steady-state (Figure 5A). When NKA block is modest (i.e., 40%) and our simulations predict regular steady-state firing activity, Na^+^ and FR nullclines cross within the FR-Na^+^ phase plot, and the fixed point of the system anchors on the stable branch corresponding to regular AP firing (Figure 9B, left panel). Conversely, with strong NKA reduction (i.e., 70%), the firing is stably suppressed (Figure 9B, right panel). Similar dynamics were observed in ventricular myocytes, where feedback of [Ca^2+^]_i_ and [Na^+^]_i_ that influence membrane voltage could explain the intermittency of early after-depolarizations [27].

### 3.3. Disruption of Na^+^ Homeostatic Processes Contributes to SAN Dysfunction in Animal Models and Patients

Our model analysis suggests that pharmacological NKA inhibition can have opposite consequences in SAN myocytes, depending on the degree of induced Na^+^ (and Ca^2+^) rise, similar to experimental observations in ventricular myocytes upon administration of cardiac glycosides [49]. Cardiac glycosides are used in treating congestive HF to promote inotropy. Moderate Na^+^ accumulation has a positive inotropic effect in ventricular myocytes and a positive chronotropic effect in our SAN cell simulations. Excessive [Na^+^]_i_ displays pro-arrhythmic side effects, including an increased propensity for spontaneous SR Ca^2+^ release and delayed after-depolarizations, and decreased lusitropy in ventricles [49], and disrupts simulated SAN pacemaker activity, which may contribute to arrhythmia due to ectopic pacemakers. Notably, patients intoxicated by cardiac glycosides also develop supraventricular arrhythmias [50]. Although cardiac glycosides decrease heart rate due to vagomimetic and anti-adrenergic effects [50], studies in isolated SAN multicellular preparations, devoid of neurohormonal control, reported an increase in FR (a phenomenon called “digitalis-induced sinus tachycardia”), development or irregular activity, and arrest of pacemaking function [46,50,51]. While our simulations reproduce these phenotypes, future experimental work should investigate whether reduction of Na^+^ load attenuates the impact of cardiac glycosides on SAM function.

Our simulations also recapitulated data from mouse models of atrial-selective NCX knockout or ankyrin-B syndrome [30,31], thus suggesting that interventions aimed at restoring Na^+^ homeostasis and/or its consequences for Ca^2+^-dependent processes can reduce the susceptibility for pacemaking irregularities. Indeed, our simulation of complete NCX block predicted loss of automaticity, as observed in isolated SAMs from NCX knockout mice [45]. Loss of function of ankyrin-B impairs targeting and stabilization of NKA, NCX, and inositol trisphosphate (InsP_3_) receptors at the transverse-tubule/SR sites in cardiomyocytes [31], leading to a broad set of cardiac dysfunctions, including impaired pacemaking [31,32,33]. Since the analysis of SAMs isolated from ankyrin-B-deficient mice suggested concomitant downregulation of NKA and NCX [32], SAN irregularities in this animal model could be facilitated by the synergistic effect described for the combined NKA and NCX block (Figure 7).

**Figure 10:**
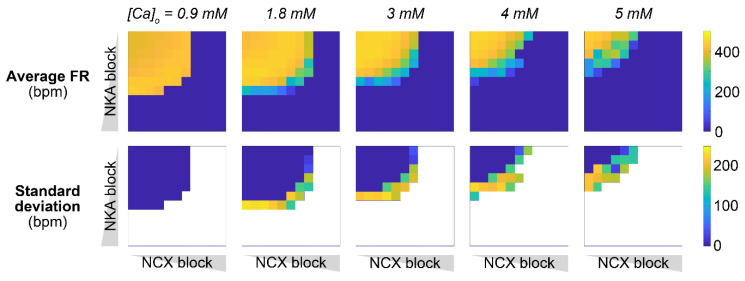
Hypercalcemia facilitates disruption of SAM automaticity. Average FR (top panels) and FR standard deviation (bottom panels) were assessed upon combinations of concomitant NKA and NCX block at different levels of extracellular [Ca^2+^]. Note that the baseline [Ca^2+^]_o_ used in our simulations is 1.8 mM.

While we simulated acute changes induced by NCX and NKA inhibition, long-term chronic changes (e.g., due to transcriptional and post-translational regulation) are likely to occur in these extremely high Na^+^ and Ca^2+^ levels. Indeed, experiments in both NCX knockout and ankyrin-B-deficient mice also revealed a ~50% decrease in I_CaL_ [29,32]. While reduced I_CaL_ is likely a mechanism to limit cellular Ca^2+^ overload [48], our analysis predicts that this maladaptive alteration further impairs SAN pacemaking by reducing the NKA and NCX block ranges that remain compatible with stable SAM function (Figure 8). Future experimental investigations could test whether increasing I_CaL_ can attenuate the pathologic phenotype observed in these mice. Notably, level of serum (or extracellular) Ca^2+^ concentration ([Ca^2+^]_o_) influences Ca^2+^ influx via I_CaL_ and the function of other Ca^2+^ handling processes in SAMs. Development of hypocalcemia and hypercalcemia have been documented in several pathologic states, and both conditions have been associated with increased pro-arrhythmic risk [52]. Our simulations show that increasing [Ca^2+^]_o_ enhances SAM automaticity (Figure 10), in agreement with recent computational work that also has suggested that this effect is even more pronounced in humans vs. small mammals [53,54]. Our results further demonstrate that increasing [Ca^2+^]_o_ increased SAM susceptibility to irregularities induced by combined NKA and NCX block (Figure 10), confirming recent experimental observations reporting that hypercalcemia not only increases FR but can also increase the propensity of SAN dysfunction in mice [55].

We assessed the translatability of our findings in mouse to human physiology by simulating the Loewe et al. model of the human SAM [53]. Human simulations confirmed our observations in murine SAMs that inhibition of NCX or NKA can disrupt SAM automaticity via Ca^2+^ overload and consequent I_CaL_ inhibition (Figure S3). As observed with our mouse model (Figure 5), preventing the accumulation of Na^+^ due to NKA block restores regular automaticity in human SAMs (see [Na^+^]_i_-clamp simulation in Figure S3C). Clamping CDI of I_CaL_ to the values predicted before block restores fast (but irregular) firing activity after disruption of human SAM function induced by NCX or NKA block (see CDI-clamp simulations in Figure S3C,D). However, the human model did not predict the chronotropic effect induced by mild NKA block in murine SAMs (Figure 4), and neither NCX nor NKA block (or their combined inhibition) led to developing the bursting activity. These results suggest that intracellular Na^+^ levels affect pacemaking function in both human and murine SAMs. However, interspecies differences likely exist in the strengths and relative roles of the processes linking Na^+^, Ca^2+^, and membrane potential homeostasis depicted in Figure 9 that warrant further investigation.

### 3.4. Limitations and Future Directions

Despite the modifications made to closely recapitulate our experimental dataset, our model maintains the same structure of the original Kharche et al. version and inherits its main limitations previously discussed in [34]. Notably, this framework does not include several components proposed to actively regulate SAM electrophysiology in control and, more so, pathologic conditions. Those include ion channels (e.g., small-conductance Ca^2+^-activated K^+^ channels [56]), transporters (e.g., InsP_3_ receptors [57], and Na^+^/H^+^ exchanger [58,59]), and intracellular signaling pathways (e.g., CaMKII- [60] and PKA-dependent cascades [61]).

Neurohumoral regulation is a major determinant of pacemaking function in vivo [62], and β-adrenergic signaling critically regulates the coupling between the membrane and Ca^2+^ clocks in human SAMs [63]. Thus, future work should investigate the role of Na^+^ homeostasis in mediating SAM response to the β-adrenergic challenge when increases in PKA-dependent phosphorylation of phospholemman enhances NKA function [64,65]. The consequent Na^+^ depletion expected in this case could, for example, be involved in the isoproterenol-induced restore of automaticity observed in dormant SAMs [63,66].

Future investigations should also extend our analysis to incorporate the structural modifications that have been associated with SAN dysfunction, including remodeling in subcellular ultrastructure [30] and functional microdomains [67] and tissue-level fibrosis [68]. Experiments in intact SAN tissue isolated from NCX knockout mice revealed bursting activity [29]. Since heterogeneous Na^+^ loading has been associated with repolarization abnormalities in ventricular tissue simulations [27], future work should explore whether intercellular [69] and inter-regional variability [44,70] affect SAN function at the tissue level.

### 3.5. Conclusions

In this study, we show that [Na^+^]_i_ dynamically modulates SAM automaticity during regular and irregular firing regimes, and reveal new mechanisms by which aberrant Na^+^ signaling plays an important role in generating cardiac disorders [71]. Experimental characterization of the mechanisms controlling SAM Na^+^ homeostasis, including testing our model predictions, may advance our understanding of cardiac pacemaking function and dysfunction and aid in identifying new therapeutic targets [72].

## 4. Methods

### 4.1. Experimental Data

Experimental data previously collected by the Proenza laboratory [35,36,37,39] were used to constrain model reparameterization. Briefly, SAMs were isolated from 2–3 months old male C58BL/6 J mice [73,74], and membrane currents and spontaneous APs were recorded at 35 °C in whole-cell and amphotericin perforated patch configurations, respectively [35,36,39]. AP characteristics (defined in Table A2) were determined for each cell from average waveforms from 5 s recording windows using the software ParamAP [40].

### 4.2. Model Development

The Kharche et al. model of murine SAMs [34], implemented in MATLAB (The MathWorks Inc., Natick, MA, USA), provided the basis for our simulations. We first modified the formulation of the following currents to match our experimental data (Figure 1A):

*(i)*  I_CaL_. The original Kharche et al. model includes isoform-specific formulations for I_CaL1.2_ and I_CaL1.3_ based on data obtained from genetically modified mice lacking either isoform. Given the lack of pharmacological agents that unequivocally distinguish between Cav1.2 and Cav1.3 when both expressed in wild-type mice, we eliminated I_CaL1.2_ and re-parameterized the formulation of I_CaL1.3_ to reproduce our experimental peak I_CaL_-voltage relationship [35]. This was obtained by negatively shifting (−7 mV) the voltage-dependence of activation and inactivation and decreasing the maximal conductance by one-third.
*(ii)*  I_to_ and I_sus_. Maximal conductances of both components of the outward K^+^ currents were increased by 2.5-fold [36].
*(iii)*  I_f_. The original I_f_ the formulation of the Kharche et al. model was replaced with our recently updated version [37]. Briefly, we modified and extended the Hodgkin-Huxley type I_f_ model originally present in the Kharche et al. framework based on our novel data in murine SAMs describing voltage-dependence of I_f_ availability and activation/deactivation kinetics. Notably, in our novel formulation, activation and deactivation of I_f_ exhibit both fast and slow kinetics [37].

Next, to reproduce the average AP properties observed in our experiments [39], we applied an established population-based optimization method [38]. We created a population of 1000 model variants by perturbing selected parameters (listed in Table A1) with random scaling factors chosen from a log-normal distribution with a median value of 1 and a standard deviation of 0.1 [75]. We assessed AP characteristics in each model in the population and then performed reverse multivariable regression [38] to identify the parameters scaling factors required to best reproduce the AP biomarkers experimentally observed (Figure 1B). We used the following set of target values: upstroke velocity of 10 mV/ms; repolarization rate of −3 mV/ms; maximum diastolic potential of −65 mV; AP amplitude of 75 mV; AP duration at 90% repolarization of 60 ms; AP duration at 50% repolarization 25 ms; cycle length of 150 ms; diastolic duration of 75 ms; diastolic depolarization rate of 100 mV/s.

### 4.3. Simulation Protocols and Analysis

Using our newly developed dataset-specific version of the Kharche et al. model [34], we simulated various degrees of reduction of NKA and NCX function by modulating their respective maximal transport rates v_NKA_ and v_NCX_, individually or in combination. In a separate set of simulations, we superimposed changes in I_CaL_ properties (i.e., ±50% in G_CaL_ and ±4 mV shift in voltage-dependence) or extracellular [Ca^2+^]. We simulated the effect of parameter perturbations for 120 s and quantified the impact on FR (average and variability) over the last 60 s. Specifically, in each simulation, we assessed the FR at 7 time points (every 10 s, from 60 to 120 s after block), analyzing the voltage signal over a time interval of 1.8 s, and then calculated average and standard deviation from the 7 samples.

To identify the subcellular mechanisms involved in regulating SAM automaticity upon NKA and NCX block, we also performed simulations in which [Na^+^]_i_ or CDI of I_CaL_ were clamped to desired values. In “CDI-clamp” simulations, we used the subsarcolemmal [Ca^2+^] signal observed in the absence of any block as input for the calculation of the changes in the state variable *Fca*, which represents the CDI-related gate in the Hodgkin-Huxley type I_CaL_ model implemented in Kharche et al. [34]. Note that *Fca*, which varies between 0 and 1, decreases as CDI increases and vice versa. Therefore, CDI values were estimated in our simulations as CDI = 1 − *Fca*. Finally, voltage-clamp simulations were performed to assess how changes in SAM automaticity influence [Na^+^]_i_ (“FR-clamp”). Specifically, the AP obtained with the updated baseline model was used as voltage command in AP-clamp simulations and stretched/compressed in time to simulate different FRs within the range of 300–600 bpm. Na^+^ levels predicted in the absence of firing activity were determined by clamping the transmembrane potential at −40 mV.

Consequences of NKA and NCX block on human SAM electrophysiology were investigated simulating the model recently developed by Loewe et al. [53].

## Data Availability

All data needed to evaluate the conclusions in the paper are present in the main text and the Supplementary Materials. Data and source codes are freely available for download at elegrandi.wixsite.com/grandilab/downloads and github.com/drgrandilab.

## Supplementary Materials

The following figures are available online at https://www.mdpi.com/article/10.3390/ijms22115645/s1.

## Appendix A

**Table A1:** List and definition of altered model parameters.

**Maximal Conductances**	
G_st_	Sustained inward Na^+^ current (I_st_)
G_Na1.1_	TTX-sensitive Na^+^ current (I_Na1.1_)
G_Na1.5_	TTX-resistant Na^+^ current (I_Na1.5_)
G_CaT_	T-type Ca^2+^ current (I_CaT_)
G_CaL_	L-type Ca^2+^ current (I_CaL_)
G_f_	Hyperpolarization-activated (funny) current (I_f_)
G_K1_	Time-independent K^+^ current (I_K1_)
G_Kr_	Rapid delayed rectifying K^+^ current (I_Kr_)
G_Ks_	Slow delayed rectifying K^+^ current (I_Ks_)
G_to_	Transient component of the 4-AP-sensitive K^+^ current (I_to_)
G_sus_	Sustained component of the 4-AP-sensitive K^+^ current (I_sus_)
G_NaB_	Background Na^+^ current (I_NaB_)
G_CaB_	Background Ca^2+^ current (I_CaB_)
**Maximal Transport Rates**	
v_NKA_	Na^+^/K^+^ ATPase (NKA)
v_NCX_	Na^+^/Ca^2+^ exchanger (NCX)
v_RyR_	Ca^2+^ release via ryanodine receptor
v_SERCA_	Sarcoplasmic reticulum (SR) Ca^2+^ pump

**Table A2:** List and definition of action potential characteristics. The values of these biomarkers were determined as described in [40].

Output		Unit
FR	Firing rate	bpm
CL	Cycle length	ms
UV	Upstroke velocity	mV/ms
RR	Repolarization rate	mV/ms
DD	Diastolic duration	ms
APD	Action potential (AP) duration	ms
APD_90_	APD at 90% of repolarization	ms
APD_50_	APD at 50% of repolarization	ms
MDP	Maximum diastolic potential	mV
AP_amp_	AP amplitude	mV
THR	Threshold potential	mV
DDR	Diastolic depolarization rate	mV/s
