# Supplementary material for: Intracellular Na+ Modulates Pacemaking Activity in Murine Sinoatrial Node Myocytes: An In Silico Analysis
Source: Int J Mol Sci. 2021 May 26;22(11):5645. doi: 10.3390/ijms22115645 (PMC8198068; doi:10.3390/ijms22115645)
Supplement: Supplementary file 1 [file ijms-22-05645-s001.zip › ijms-1209185-supplementary.pdf]

Supplementary Materials

Intracellular Na^+ modulates pacemaking activity in murine sinoatrial node myocytes: an *in silico* analysis

Stefano Morotti,[1,@] Haibo Ni,[1] Colin H. Peters,[2] Christian Rickert,[2] Ameneh Asgari-Targhi,[1] Daisuke Sato,[1] Alexey V. Glukhov,[3] Catherine Proenza,[2,4] and Eleonora Grandi[1,@]

[1] Department of Pharmacology, University of California Davis, Davis, CA 95616, USA

[2] Department of Physiology and Biophysics, University of Colorado Anschutz Medical Campus, Aurora, CO 80045, USA

[3] Department of Medicine, Cardiovascular Medicine, University of Wisconsin Madison School of Medicine and Public Health, Madison, WI 53705, USA

[4] Department of Medicine, Division of Cardiology, University of Colorado Anschutz Medical Campus, Aurora, CO 80045, USA

[@] Correspondence:

Stefano Morotti
smorotti@ucdavis.edu

Eleonora Grandi
ele.grandi@gmail.com

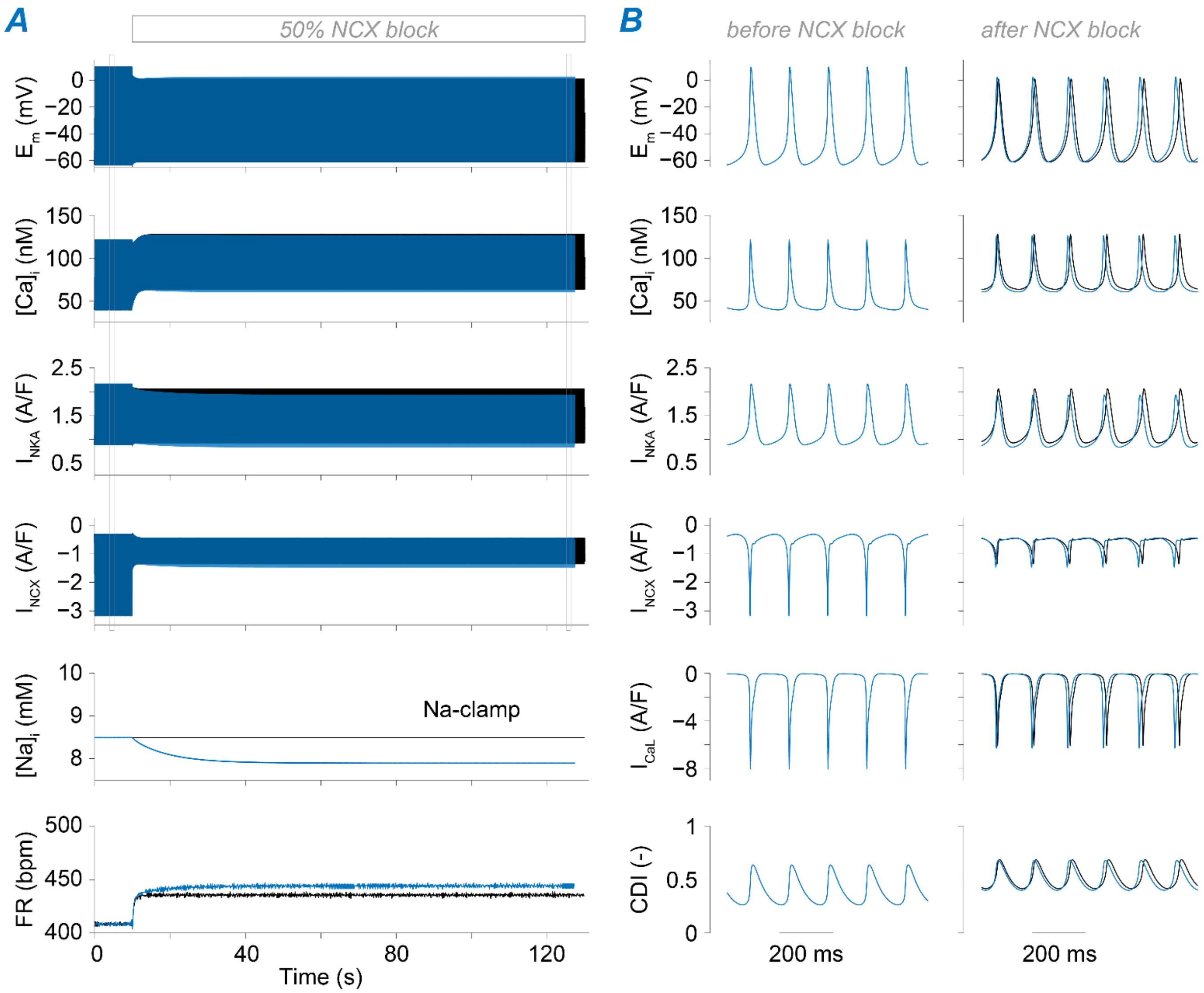

Figure S1. Consequences of 50% block of NCX. (**A**) Time course of membrane potential, $[Ca^{2+}]_i$, NCX current, $[Na^+]_i$, and FR predicted upon sudden 50% block of NCX maximal transport rate (at t = 10 s). Black traces are obtained clamping $[Na^+]_i$ to the initial value, while blue traces are obtained simulating the normal condition in which $[Na^+]_i$ is free to change. (**B**) Comparison between the time course before applying the block and at the end of the simulation for membrane potential, $[Ca^{2+}]_i$, NCX current, NKA current, I_{CaL}, and its Ca^{2+}-dependent inactivation. CDI values were calculated from the state variable *Fca*, which represents the gate describing CDI in the Hodgkin-Huxley type I_{CaL} model in the Kharche et al. framework (CDI = 1 – *Fca*, with *Fca* varying from 0 to 1).

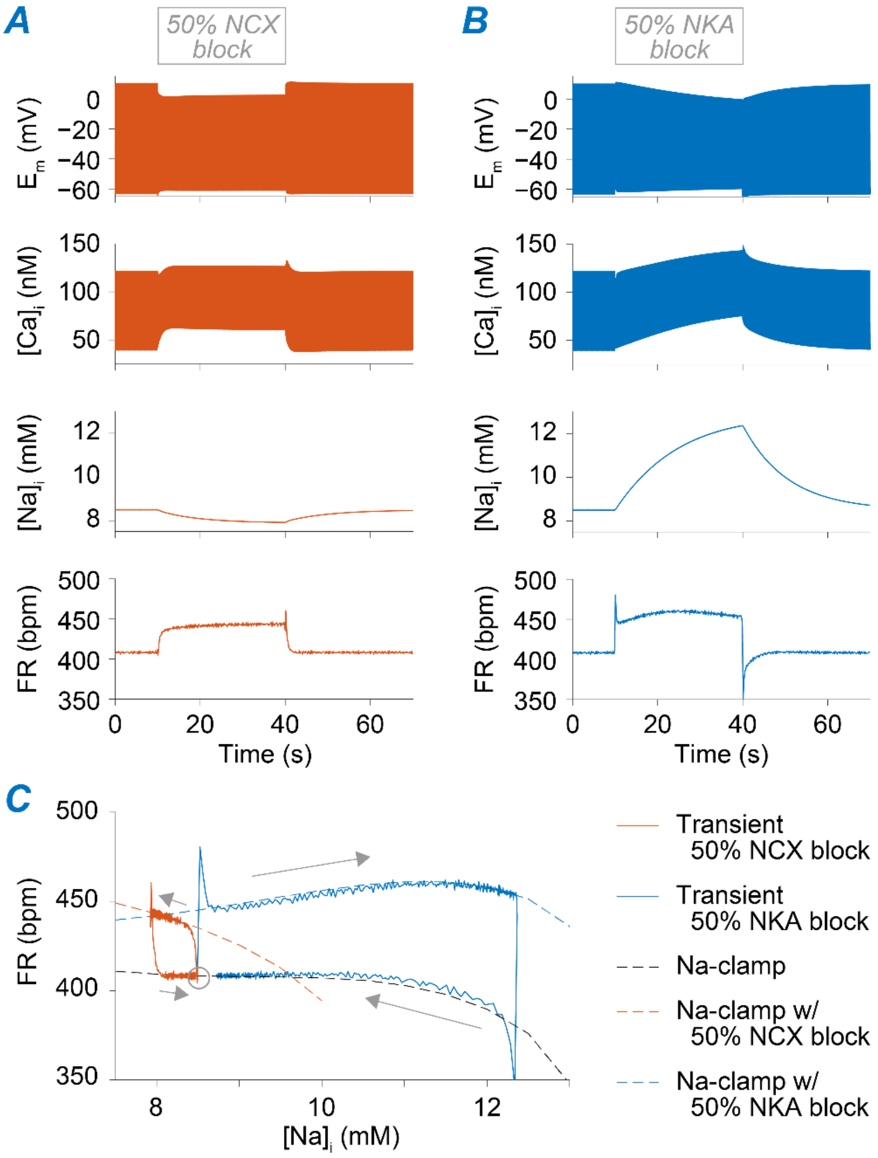

Figure S2. Consequences of transient 50% block of NCX or NKA. Time course of membrane potential, $[Ca^{2+}]_i$, $[Na^+]_i$, and FR predicted upon 50% block of NCX (**A**) or NKA (**B**) simulated between $t = 10$ s and $t = 40$ s. Panel **C** shows FR-$[Na^+]_i$ phase plots obtained in both simulations, together with FR values predicted when clamping $[Na^+]_i$ at the indicated levels with standard model parameters (black dashed line) or upon 50% block of NCX (orange dashed line) or NKA (blue dashed line). The grey circle corresponds to the initial condition, and the arrows indicate the direction of changes over time.

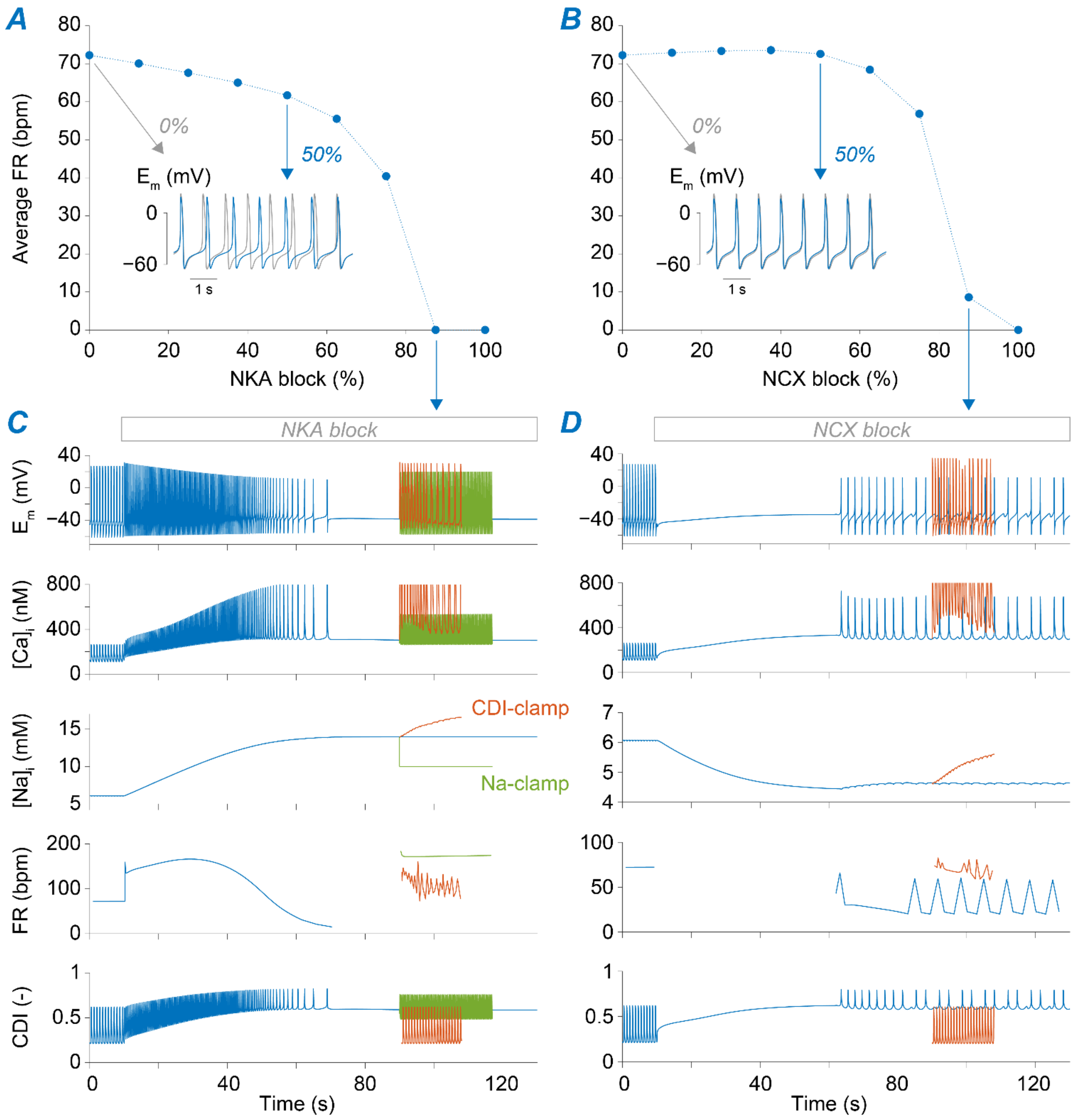

Figure S3. Consequences of NKA and NCX block on human SAM electrophysiology. Panels **A** and **B** report average FR values determined simulating various extents of NKA or NCX block with the Loewe et al. model of human SAMs. Insets show voltage traces obtained simulating control condition and 50% block. Panels **C** and **D** report time courses of membrane potential, $[Ca^{2+}]_i$, $[Na^+]_i$, FR, and Ca^{2+}-dependent inactivation of I_{CaL} predicted upon 87.5% block of NKA or NCX (at t = 10 s). Green traces are obtained by clamping $[Na^+]_i$ to 10 mM after t = 90 s; orange traces are obtained by imposing (after t = 90 s) the values of CDI predicted before block; blue traces are obtained simulating the model without any constrain on $[Na^+]_i$ or CDI. CDI values were calculated from the state variable *Fca* in the Loewe et al. model (CDI = 1 – *Fca*).
